# Mediterranean Diet Effects on Type 2 Diabetes Prevention, Disease Progression, and Related Mechanisms. A Review

**DOI:** 10.3390/nu12082236

**Published:** 2020-07-27

**Authors:** Sandra Martín-Peláez, Montse Fito, Olga Castaner

**Affiliations:** 1 Department of Preventive Medicine and Public Health, Faculty of Medicine, University of Granada, 18071 Granada, Spain; sandramartin@ugr.es; 2 Instituto de Investigación Biosanitaria (ibs.GRANADA), 18071 Granada, Spain; 3 Cardiovascular Risk and Nutrition Research Group, Hospital del Mar Medical Research Institute [IMIM], 08003 Barcelona, Spain; mfito@imim.es; 4 Consorcio CIBER, M.P. Fisiopatologia de la Obesidad y Nutrición [CIBERObn], Instituto de Salud Carlos III [ISCIII], 28029 Madrid, Spain

**Keywords:** quality diet, primary prevention, diabetes, Mediterranean, mechanisms

## Abstract

The search for a quality diet has grown over the past decade. Diet is considered one of the pillars for the prevention and progression of several diseases, among them: diabetes. Type 2 diabetes (T2D) is an epidemic of western countries that increases the vulnerability of other diseases, such as cardiovascular and cancer. T2D is associated with lifestyle and diet. The traditional Mediterranean diet has proven its benefits over several cardiovascular risk factors, and specifically on diabetes. This review compiles recent published evidence on the effects of the Mediterranean diet on the incidence and progression of type 2 diabetes (T2D) and its relation with several other cardiovascular healthy diets. We will also focus on how the Mediterranean diet could play a role in T2D-related mechanisms, such as anti-inflammatory or antioxidant compounds, glucagon-like peptide agonist compounds, and changes in gut microbiota. Each component of the Mediterranean diet could be involved in processes related to diabetes homeostasis, many of them sharing common physio-pathological pathways. The importance of this diet within the set of habits of a healthy lifestyle must be emphasized.

## 1. Background of Diabetes Epidemiology

At present, approximately 425 million adults suffer from diabetes which caused four million deaths globally in 2017 [1]. Its prevalence in Spain is around 13.8% in adults and it is more common in men [2]. These figures, which have reached epidemic dimensions, signify that the prevention and treatment of diabetes are currently one of the main objectives of public health policies. Moreover, by 2035, the worldwide prevalence is estimated to affect 592 million individuals (10.1% of the global population) [3]. Such a trend has been confirmed by the International Diabetes Federation (IDF) with its own estimation of diabetes affecting 700 million by 2045 [4]. There is, therefore, a need for preventive activities to reduce the ongoing progress of this disease and its associated complications [5]. Furthermore, patients with diabetes are considered a vulnerable population due a diversity of comorbidities. Their life expectancy is shortened by approximately six years [6], in part due to the fact that diabetes doubles the risk of cardiovascular disease [7,8] and increases the risk of other diseases such as cancer [6,9,10]. A meta-analysis published in 2011 studied the associations between diabetes and mortality due to a number of factors including several types of cancer, such as liver, pancreas, ovarium, colorectal, lung, breast, and biliary [6]. In addition, diabetes is associated with cognitive impairment [11], renal or hepatic failure, mental conditions, digestive diseases, pneumonia, and others [6]. It is also linked to macro- and micro-vascular complications, including diabetic retinopathy, peripheral arteriopathy, renal diabetic disease, and diabetic neuropathy, all of which affect the patient’s health and quality of life. 

## 2. Diabetes and Lifestyle

The IDF has confirmed that even though the etiology of type 2 diabetes (T2D) is not completely defined, it is known that individuals with overweight, unhealthy diets, sedentarism, and a family history of diabetes have a major risk of developing this condition [12]. Unhealthy lifestyles can promote the presence of diabetes. Therefore, steps to improve them are one of the pillars of its prevention and treatment at pre-illness stages. Research into lifestyle-related behavior regarding the modification of unhealthy diets, and promotion of better dietary patterns associated with a decrease in T2D incidence [13] is therefore extremely relevant. In recent years, a considerable number of studies analyzing the benefits of diet on the incidence of diabetes have been published [13,14,15]. The most recent reviews and meta-analyses compare low-fat or low-carbohydrate diets, Mediterranean-type diets, and vegetarian or vegan diets [14], all of which are considered healthy dietary patterns compared to western, cafeteria-based ones or those that do not integrate any type of intervention [15]. As a novelty, recent studies have also assessed other types of interventions such as mobile applications and cooking workshops [16].

## 3. Mediterranean Diet Lifestyle

The traditional Mediterranean diet is characterized by cooking seasonal and local products, and enjoying socialization with meals. It consists of a daily abundance of vegetables, a variety of minimally processed whole grain bread, and other cereals and legumes as the staple food, nuts and seeds, fresh fruit as the typical daily dessert; sweets based on nuts, olive oil, and honey consumed only during celebratory occasions; cold pressed extra-virgin olive oil (EVOO), nuts and seeds as the principal source of fat; a low to moderate consumption of dairy products (mainly local cheese and yogurt) consumed in low amounts; a moderate consumption of fish, poultry, and eggs, a low consumption of red meat (once a week approximately), and a moderate consumption of wine, normally with meals [17,18]. 

Regular physical activity is also part of the Mediterranean lifestyle, influenced by the climate.

## 4. Mediterranean Diet and Other Healthy Diets Effects on the Incidence and Control of T2D

We conducted a structured literature search at the United States National Library of Medicine, National Institutes of Health, and PubMed, considering publications released over the past five years which included a systematic review or meta-analysis regarding the effect of the Mediterranean diet on the incidence or better control of T2D (Table 1). The selected reviews covered more than 100,000 individuals participating in clinical trials and prospective cohort studies. Most of the reviews analyzed the association of T2D incidence with the consumption of a Mediterranean diet and sometimes compared it to other healthy diets. To assess Mediterranean diet adherence, most studies used a Mediterranean diet score. That is to say, the sum of a few items obtained from a food intake questionnaire with emphasis on the type and amount of food consumed based on the food pyramid.

A major part of these studies demonstrated an association between adherence to dietary patterns and a decrease in T2D risk [13,14,15,19]. Regarding the analysis of incidence based on the degree of adherence to the Mediterranean diet, the studies showed a reduction of approximately 20% in T2D risk with a high score on the Mediterranean Diet Adherence Questionnaire [14,19,20]. Specifically, a meta-analysis involving eight cohort studies with a total of 122,810 individuals found that higher adherence to the Mediterranean diet was associated with a 19% lower risk of suffering T2D, highlighting the long-term protective effects of the Mediterranean diet [14]. This study analyzed whether diet protection against diabetes appeared to be more marked in European populations than North American ones. It also highlighted the long-term protective effect of the Mediterranean diet, referring to follow-up studies of more than 10 years [14].

Koloverou and colleagues included in their meta-analysis one clinical trial and nine prospective studies. They observed that the T2D risk was reduced by 23% among individuals who had a maximum or minimum adherence questionnaire score in the Mediterranean diet [19].

In terms of data provided by clinical trials, the PREDIMED study, a large-scale, multicenter, controlled randomized trial, reported that a Mediterranean diet enriched with EVOO or nuts prevented diabetes, as compared to a low-fat diet, reducing the risk by 52% in the elderly with a high cardiovascular risk [20]. This beneficial effect was mainly attributed to the overall composition of the dietary pattern and not to caloric restriction, increased physical activity, or weight loss [20].

In 2017, a systematic review and meta-analysis [13] comparing different dietary patterns in a population of approximately 1.5 million participants also described that both the Mediterranean diet and other cardio-healthy diets reduced the risk of diabetes. One of these was the DASH (Dietary Approaches to Stop Hypertension Diet) [21] which aimed to control sodium intake (between 1500 milligrams (mg) and 2300 mg of sodium/day) and promote the consumption of vegetables, fruit, and low-fat dairy products, as well as moderate amounts of whole grains, fish, poultry, and nuts. Another measure was the AHEI (Alternative Healthy Eating Index) which assessed adherence to American dietary guidelines and the food pyramid. The review focused on concordant food-based groupings and yielded results of decreasing diabetes incidence related to another meta-analysis of prospective cohort studies [22] in which both the DASH and the Mediterranean diet maintained a 20% reduction in the risk of T2D. In this analysis, no differences were found comparing the two types of diets, as well as the follow-up time and geographical effect. The meta-analysis observed that although diets associated with the prevention of T2D may vary in composition, there are several common components such as table oils (olive oil) and whole grains, fruit, nuts, vegetables, legumes, protein-rich foods [e.g., white meat and seafood], moderate alcohol consumption, and reduced intake of red/processed meat and sweetened beverages [22].

Apart from the DASH and AHEI diets, the Mediterranean diet has also been compared to other diets, especially low-fat, low-carbohydrate, paleolithic, and vegetarian/vegan ones.

The paleolithic diet consists of eating fruit, vegetables, nuts, seeds, lean meat (especially from pasture-fed animals), fish rich in omega-3 fatty acids, and olive/walnut oils. There is, however, little scientific evidence regarding the general effects of this diet and less with respect to T2D incidence [23]. In fact, the literature about any of the alternative diets (vegan, semi-vegetarian, etc.) is not very extensive [24].

The vegetarian diet is characterized by the consumption of whole plant foods and encompasses a variety of diets that include one or another group of foods. The most restrictive is veganism which consumes no animal-derived products; lacto-ovo-vegetarians do not eat meat, but regularly consume milk, dairy products, and eggs; fish-vegetarians consume fish, milk, dairy products, and eggs; and semi-vegetarians consume meat and meat products minimally but regularly. 

It has been observed that vegetarian diets are inversely associated with the risk of developing T2D regardless of the positive association of meat consumption with its development. With respect to follow-up studies, a publication from an Adventist society referred to a 74% reduction in diabetes incidence from a meat-based diet in a 17-year follow-up [25]. Another study in a Buddhist society found that adherence to a vegetarian diet was associated with a 35% lower risk of developing diabetes [26]. In addition, the Rotterdam study [27] showed that a vegetarian-based diet adapted from Satija and colleagues reduced the risk of diabetes (~18%) and pre-diabetes (~11%) after a follow-up of four to seven years.

Returning to the Mediterranean diet, there is a considerable number of publications regarding its specific components, some of which have been individually reported to reduce diabetes incidence. For instance, a higher intake of olive oil was associated with a lower risk of diabetes in a cohort of women during a 22-year follow-up [28]. The authors explained that substituting other types of seasonings (e.g., margarine, butter, and mayonnaise) for olive oil was inversely associated with the onset of the disease [28].

## 5. Benefits of the Mediterranean Diet on Glycemic Control

Over the years, a number of diets have been proposed as being suitable for diabetic patients without any clear conclusions [29,30]. According to the recommendations of the American Diabetes Association (ADA), nutritional therapy for adults with diabetes should focus on promoting healthy eating patterns based on key nutrients, varied, selected, and integrated in the right amount, aiming to maintain a healthy weight, and reach optimum levels of glycosylated hemoglobin (HbA1c), blood pressure, and lipid profile. To achieve this, the ADA emphasizes that cultural preferences should be considered, as well as the areas where patients live, access to recommended foods, and a willingness to change [31]. It refers to maintaining the pleasure of eating and providing the necessary tools to empower patients to establish healthy eating patterns themselves, rather than talking about unique foods or micro/macronutrients. In this context, a healthy dietary pattern such as the traditional Mediterranean one could be the key to obtaining a proper control of diabetes [31,32].

Articles published in indexed journals rely on different parameters to analyze improvement in diabetes control. They are generally based on enhancements in HbA1c levels, and, in some cases, on basal glycemia. A systematic review of eight meta-analyses and five randomized controlled trials examined the effect of the Mediterranean diet on the treatment of diabetes and prediabetic states [33]. It indicated that in diabetic patients, adherence to the Mediterranean diet was associated with lower HbA1c levels and a better profile of cardiovascular risk factors, compared to control diets, mainly with a low-fat diet content [33].

The success of the Mediterranean diet in reducing HbA1c values is clear; nevertheless, the degree to which it is compared depends on the diet. For example, if we compare it with a low-fat one [14,22] or a control group [34], it seems that the Mediterranean diet reduces HbA1c by 0.32 to 0.53 percentage units. In contrast, when compared to other diets, such as the vegan or paleolithic ones, two meta-analyses did not show an improvement in the reduction of HbA1c [14,30]. On the other hand, in patients with newly diagnosed diabetes, another clinical trial described better glycemic control in a group adhering to a Mediterranean diet with energy restriction compared to those that followed a low-fat diet [34].

Good control of glycemia can reduce the risk of diabetes complications in the short-, medium-, and long-term [35]. The ATTICA study described that adherence to the Mediterranean diet was linked to improved fasting glucose homeostasis, insulin levels, and a better insulin resistance index (HOMA) in both normoglycemic individuals and diabetic participants. Those who had a high score of Mediterranean diet adherence presented 15% lowered basal glucose and insulin, and a 27% increased HOMA index [36]. In this regard, in the 722 PREDIMED participants at high cardiovascular risk, a decrease of 0.39 and 0.30 mmol/L in fasting glycemia was observed in the oil-enriched and nut-enriched diet groups, respectively, with respect to the control in the absence of weight loss after three months of intervention [37]. In the same line, two randomized trials assessed the beneficial effect of the Mediterranean diet on glycemia. One trial with 279 participants in a six-month intervention with a Mediterranean diet pattern reported a reduction of 0.4 percentage units in HbA1c, compared to the control [38]. The other with 322 participants randomized to three different interventions (low carbohydrate and without calorie restriction; Mediterranean hypocaloric; low fat and hypocaloric) found concordant results. In a subgroup of 36 individuals with diabetes, there was also a reduction of HbA1c of 0.4 ± 1.3% in the low-fat diet group, 0.5 ± 1.1% in the Mediterranean diet group, and 0.9 ± 0.8% in the low-carb diet group. Finally, in a one-year randomized trial of 259 patients with T2D, three diets were compared, low-carbohydrate Mediterranean, traditional Mediterranean, and an ADA-proposed one [39]. The mean weight loss was 10.1, 7.4, and 7.7 kg, respectively, and reductions in HbA1c were described in participants assigned to the low-carbohydrate Mediterranean diet and the traditional Mediterranean one (0.4% and 0.2% reductions, respectively) versus the ADA diet [39]. Despite the promising results of these studies, glycemia levels rise in the course of T2D [40,41]. This implies the need for a sequential increase in therapies [41]. In this regard, in a randomized trial of 215 patients with newly diagnosed T2D who were assigned to a low-carbohydrate Mediterranean-style diet, there was a delay in the need for new hypoglycemic drugs with respect to a low-fat diet, yet 44% to 70% of participants required treatment after a four-year follow-up [42]. It should be noted that participants assigned to the Mediterranean-style diet lost more weight than those on the low-fat one [42].

Apart from the general Mediterranean diet pattern, the scientific community has investigated the effect of certain specific components such as olive oil. In a randomized, cross-over trial it was observed that regular and moderate daily consumption (25 mL/day) of virgin olive oil (phenolic compounds: 577 mg/kg) for eight weeks in overweight and T2D patients lowered fasting plasma glucose and HbA1c [43]. Another study examined how monounsaturated fatty acids, the main component of olive oil, were associated with a lower concentration of fasting plasma glucose in 4903 Italian men and women aged 20 to 59 years [44,45]. In a cross-sectional study in Spain (PIZARRA), insulin resistance was found to be lower in individuals who consumed olive oil compared to those who consumed sunflower oil or a combination [3].

With regard to other diets, we find that there is very little scientific evidence regarding the paleolithic diet effect on the treatment and control of diabetes. Few clinical trials have been published and the sample sizes are limited (14–32 individuals). It seems that in a clinical trial where T2D participants underwent 12 weeks of paleolithic diet compared to a regular one, there was an improvement in diabetes management; nevertheless, this should be corroborated with larger sample studies [23].

The same goes for publications regarding the effect of vegetarian diets on the treatment of established diabetes. They refer to studies that are quite small when comparing a vegetarian diet in general, or some subtype with the usual diet prescribed for diabetic individuals. A number of authors have differentiated between these types of diets while others have grouped them. In general, however, they are considered beneficial for both the prevention and control of diabetes [46,47,48].

In brief, there is good evidence that adherence to the Mediterranean diet seems to have a protective role in glycemic control, reducing HbA1c, and lowering fasting levels in addition to decreased insulin resistance and mortality. Sleiman et al. suggest in their review that reducing oxidative stress, inflammation, and insulin resistance are all possible mechanisms by which the Mediterranean diet pauses as a protective dietary pattern [49].

## 6. Mechanisms Involved in Mediterranean Diet Effects on T2D

The current knowledge about the mechanisms developing diabetes indicates that they are varied and interconnected. The main physio-pathological process of T2D is the state of sustained hyperglycemia caused by pancreatic β-cells impaired insulin secretion and/or cell insulin resistance (IR). This signifies that either insulin is not generated in adequate amounts, or it is unable to get glucose into the cells, or both. In any case, the consequences are the dysregulation of carbohydrate, lipid and protein metabolism, and eventually macro- and micro-vascular complications [50]. Proper insulin secretion by β-cells can be influenced by several factors in addition to genetic abnormalities or aging [51]. In this regard, lipotoxicity [52], glucotoxicity [53], reactive oxygen stress [54], activation of inflammatory pathways [52], IR leading to β-cell stress [52,55], and/or the decrease in incretin effect (GPL1 and GIP) on β-cells, amongst others, contribute to β-cell impaired insulin secretion. How cells become resistant to insulin is a major area of research. Currently, it is known that there exist both intrinsic cellular pathways and extrinsic mechanisms. The first include cellular mitochondrial dysfunction, oxidative stress, and endoplasmic reticulum stress. The second comprise adipocyte and fatty acid level alterations and inflammation [56].

### 6.1. Mediterranean Diet and T2D

Although T2D is a multifactorial disease which involves genetic and environmental factors, healthy lifestyles, including proper diet, are considered key factors in its development [57]. We will therefore focus on how the Mediterranean diet could play a role in T2D-related mechanisms.

As previously stated, many studies have found a positive effect of the Mediterranean diet on T2D. In order to elucidate which mechanisms are behind these effects it is necessary to examine its characteristics.

The Mediterranean diet is able to reduce central obesity and, in turn, reduce obesity-related chronic diseases [58] such as T2D. In fact, this diet is superior to low-fat ones for long-term weight loss [59]. In addition, it is associated with a more significant improvement of IR in obese individuals when compared to other low-energy dietary approaches. Therefore, the composition of the Mediterranean diet, rather than the calories provided, must play a role on T2D positive effects. Moreover, while the individual effects of the distinct dietary components may be too small to be detected, their additive impact may be large enough to be discerned [18].

### 6.2. Mechanisms Based on Anti-inflammatory/Antioxidant Compounds

The effects exerted by the Mediterranean diet on T2D could probably be attributed to its anti-inflammatory/antioxidant compounds. It is known that individuals with diabetes have significantly lower levels of ascorbic acid, β-carotene, and α-tocopherol/cholesterol ratio than non-diabetic ones [60]. The consumption of this diet raises plasma levels of diet-derived plasma antioxidants [61], increases both the plasma ferric reducing antioxidant potential (FRAP) and the total radical-trapping antioxidant parameter (TRAP) [62], lowers c-reactive protein (CRP) levels [63], and prevents the acute hyperglycemia effect on inflammation, oxidative stress, and endothelial function.

Of the vast variety of anti-inflammatory/antioxidant compounds present in the Mediterranean diet, the phenolic compounds (PC) are the most ubiquitous, particularly flavonoids. The flavonoid quercetin is able to activate the insulin-independent adenosine monophosphate-activated protein kinase (AMPK) pathway of skeletal muscle cells, slowing the oxygen consumption of adenosine diphosphate in isolated mitochondria [64]. In addition, quercetin can enhance the uptake of glucose in skeletal myocytes through an AMPK-dependent up-regulation of glucose transporter GLUT-4 under oxidative stress conditions [65].

In addition to olive oil, PC are found in fruit, vegetables, legumes, and cereals. The major PC in olive oil are oleuropein, hydroxytyrosol, and tyrosol, and they are able to exert antioxidant and anti-inflammatory activities [66]. In this regard, it has been observed that the PC in EVOO improve oxidative stress by decreasing the activation of pro-inflammatory mediators and increasing bioavailability of nitric oxide, which enhances the vasodilator-dependent response of endothelium [67].

Apart from PC, EVOO is rich in poly-unsaturated fatty acids (PUFA) which ameliorate the adipose tissue inflammatory responses, providing beneficial effects on insulin sensitivity [68]. In obesity, dysfunctional adipose tissue overproduces pro-inflammatory cytokines and chemokines, such as tumor necrosis factor alfa, interleukin-6, and resistin, which activate intracellular pathways that trigger IR in insulin-target tissues. Thus, the anti-inflammatory potential of PUFA may indirectly improve peripheral insulin responsiveness, reducing the risk of glyco-metabolic alterations in patients with IR [68]. In this regard, the monounsaturated fatty acids (MUFA), the main fat of EVOO (especially oleic acid) and also present in other components of the Mediterranean diet, are thought to counteract the effect promoted by saturated fatty acids (SFA) which decrease the insulin sensitivity (IS) of peripheral tissues [69,70]. Moreover, IS and β-cell function progressively improve in the postprandial state as the ratio of MUFA to SFA in the diet increases [71]. Besides the beneficial effects of EVOO PUFA on inflammation and oxidation, favorable effects can be observed only at a total fat intake below 37% of energy, since a higher fat intake increases the risk of IR irrespective of quality [72].

EVOO also contains other potent antioxidants such as α-tocopherol, carotenoids, and phytosterols [73]. Furthermore, it has been shown that 50 g of newly pressed EVOO contains up to 9 mg of olechantal, a phytochemical with ibuprofen-like COX-inhibitory activity [74]. The Mediterranean diet is also a rich source of omega-3 fatty acids whose intake is inversely correlated with circulating inflammatory markers and triglyceride levels. The anti-inflammatory effects of omega-3 fatty acids seem to be mediated by binding to the G-protein-coupled receptor 120 and inhibition of NLRP3 inflammasome activity [75,76].

### 6.3. Mechanisms Based on Glucagon-like Peptide Agonist Compounds

The glucagon-like peptide (GLP-1) is an incretin hormone which promotes insulin generation and secretion, and inhibits gastric emptying and glucagon secretion, thus having a favorable effect on the management of T2D [77]. GLP-1 has been shown to improve endothelial function in diabetes [78,79] possibly by increasing the antioxidant defenses of the endothelium [80] and decreasing oxidative stress generation [79]. In addition, it may influence satiety at the central nervous system level, attenuating appetite sensations and, consequently, the amount of food consumed so that energy intake does not exceed expenditure. It has been suggested that hyperglycemia induces GLP-1 resistance, mainly through the generation of oxidative stress [79]. Eventually, β-cell resistance to GLP-1 contributes to progressive failure in the function of β-cells. The Mediterranean diet improves the action of GLP-1 action. In particular, PUFA from EVOO may have shown to bind and stimulate G-protein-coupled receptors such as GPR120, leading to an increased secretion of GLP-1 from enteroendocrine L-cells [80]. By stimulating insulin release from pancreatic β-cells, with immediate consequences of increased glucose uptake from skeletal muscles, raised GLP-1 levels may in turn limit postprandial hyperglycemia [81].

### 6.4. Mechanisms Based on Branched Chain Aminoacid Management

Branched chain amino acids (BCAA) play critical roles in the regulation of energy homeostasis, nutrition metabolism, gut health, immunity, and disease. Moreover, current evidence supports BCAA, and their derivatives, as potential biomarkers of pathologies such as IR, T2D, cancer, and cardiovascular diseases all of which are closely associated with BCAA catabolism and balance [82]. High levels of serum BCAA (valine, leucine, isoleucine) are positively associated with increased T2D risk [83]. Elevated levels of BCAAs are known to activate mTOR complex 1 (mTORC1) which leads to IR through the phosphorylation of insulin receptor substrate 1 (IRS-1) [84,85]. BCAA also stimulate the activation of the redox-sensitive transcription factor NF-κB, resulting in the release of pro-inflammatory molecules (IL-6, TNF-α, intracellular adhesion molecule-1, CD40L) and the migration of peripheral mononuclear blood cells [86]. These pro-inflammatory changes could contribute to the development of IR. In a large-scale randomized trial, the regular consumption of EVOO has been reported to decrease the levels of plasma BCAA [87].

### 6.5. Mechanisms Based on Changes in Gut Microbiota

Gut microbiota is composed mainly of bacteria, although it also includes commensal populations of fungi, viruses, archaea, and protists. Regarding bacteria, there are four main phyla living in the gut microbiota (*Firmicutes*, *Bacteroidetes*, *Actinobacteria*, and *Proteobateria*) each of which contain a vast variety of bacterial strains. When living in harmony, these bacteria exert fundamental roles in human physiology and metabolism. In contrast, gut microbiota imbalance, or dysbiosis, can negatively affect human homeostasis and is the key in the pathogenesis of a variety of illnesses including T2D [88,89]. For example, T2D has been related to decreases in *Firmicutes* [90] and other butyrate-producing species [91,92], and to increases in opportunistic pathogens [93] and sulfate-reducing bacteria [91,92]. Moreover, increased levels of BCAA serum from IR-individuals correlate with a gut microbiome with enriched biosynthetic potential for BCAA. *Prevotella copri* and *Bacteroides vulgatus* have been identified as the main species driving the association between biosynthesis of BCAA and IR [94]. Dysbiosis can lead to increased gut permeability and intestinal Toll-like receptor (TLR) activations through microbial components and pathogen-associated molecular patterns (PAMPs) including lipopolysaccharide (LPS), flagellin, lipoteichoic acid, and peptidoglycan resulting in the production of inflammatory cytokines [95]. In fact, bacteria lipopolysaccharide levels are higher in diabetic compared to non-diabetic patients [96]. These alterations also have consequences on the microbial products generated by bacterial metabolism which, in turn, have an impact on T2D. For example, a reduction in the excretion of the microbial metabolites hippurate, PAG and p-cresol, as well as trigonelline and 3-hydroxymandelate, has been observed in T2D patients [97].

Diet content and quantity play a major role in shaping the human microbiota composition and function [98,99]. Complex interactions between nutrients and microorganisms dictate beneficial or detrimental outcomes to host health [100]. In this regard, the Mediterranean diet has been observed to positively influence gut microbiota. For example, obese men consuming this diet for one year obtained a protective effect on the development of T2D through different specific changes in the gut microbiota [101]. The Mediterranean diet is rich in dietary fiber and complex carbohydrates. In this regard, the main gut microbiota microbial products, the short chain fatty acids (SCFA), are generated by fermentation of dietary fibers. SCFA (acetate, butyrate, and propionate) exert many relevant functions for the host. Regarding T2D, SCFA contribute to the regulation of glucose and lipid metabolism by the activation of SCFA receptors in the liver, adipose tissue, brain, and pancreas [101]. In addition, SCFA can stimulate GLP-1 and GLP-2 secretion, thereby enhancing insulin sensitivity, pancreatic β-cell proliferation [102], and satiety [103]. In this regard, in T2D patients, a deficiency in SCFA [104] has been observed, particularly a reduction in butyrate [105]. The Mediterranean diet can stimulate the production of SCFA by gut microbiota due to its elevated content in dietary fibers. Other metabolites with potent anti-inflammatory and antioxidant properties generated by gut microbiota metabolism, such as Indole-2 propionic acid (IPA), have been reported to increase after four days of ingestion of the Mediterranean diet compared to four days of fast food [106].

## 7. Conclusions

To sum up, there is consistent evidence regarding the inverse association between the adherence to a Mediterranean diet and incidence of T2D. In addition, some evidence of the association between DASH diet and T2D exists. Furthermore, the Mediterranean diet has been shown to decrease HbA1c levels compared to a control group (such as low-fat diet and low-carbohydrate diet). On the other hand, vegan and low glycemic index diets also improve HbA1c levels.

To sum up, each component of the Mediterranean diet could be involved in processes related to diabetes homeostasis, many of them sharing common physio-pathological pathways. The Mediterranean diet adherence could play a role on T2D-related mechanisms, such as anti-inflammatory/antioxidant actions, glucagon-like peptide agonist compounds, and changes in gut microbiota. Overall, single actions from different nutrients and derivative metabolites could be enhanced by interactions and synergies that make the Mediterranean diet an invaluable tool in the primary and secondary prevention of diabetes. The importance of this diet within the set of habits of a healthy lifestyle must be emphasized.

## Figures and Tables

**Table 1:** Systematic reviews and meta-analysis of the association between Mediterranean diet and type 2 diabetes (T2D).

First Author; Year	Type of Study	Type of Intervention	Population	Follow-up	Quality of the Studies	Output [s]	Results
Jannasch, F.; 2017	SR, MA 48 prospective studies: 18 cohorts/ACRs	MD, DASH, AHEI	Non-diabetic healthy adults *n* ≈ 1.5 millions	4.1–23 years	27 high quality 21 acceptable (Scottish Intercollegiate Guidelines Network checklist)	Incidence T2D	Adherence to MD (RR quantiles: 0.87; 95% CI: 0.82, 0.93), DASH (RR: 0.81; 95% CI: 0.72, 0.92), and AHEI (RR: 0.79; 95% CI: 0.69, 0.90) associated with a decreased risk of T2D.
Schwingshackl, L.; 2015	SR, MA 1 RCT, 8 prospective studies	MD	Healthy adults or with CV risk factors *n* = 122,810	3.2–20 years	Quality moderate	Incidence T2D	Adherence to MD high vs. low. RR: 0.81; 95% CI 0.73, 0.90, p < 0.0001 associated with a decrease in T2D incidence.
Koloverou, E.; 2014	SR, MA 1 RCT, 9 prospective studies	MD Control diet	Healthy adults with or without CV/T2D *n* = 136,846	3.5–14 years	Publication bias	Incidence T2D	High adherence to MD was associated with a 23% decreased risk of T2D, comparing the highest vs. lowest punctuation of MD RR = 0.77, 95% CI: 0.66, 0.89.
Esposito, K.; 2014	MA 8 prospective studies, 30 cohorts	MD DASH	Adults >20 y *n* = 21,372	3.2–23 years	High heterogenicity. No publication bias. Quality score >7 (0 to 9)	Incidence T2D	Healthy diet RR: 0.80 (95% IC 0.74–0.86). MD vs. DASH: no changes in incidence of T2D.
Esposito, K.; 2015	SR 8 MA, 5 RCTs	MD Control diet	Adults with T2D or at risk *n* = 2087	>6 months	MA: moderate RCTs: low	Incidence T2D Glycemic control	MD decreases HbA1c in 0.3–0.47% compared to low fat-diet. High MD adherence decreases T2D by 19–23%.
Schwingshackl, L.; 2018	SR, MA 56 RCTs	Low-fat diet or vegan, MD, LC, paleolithic hyperprotein diet	Adults with T2D *n* = 4937	3–48 months	Low, moderate credibility	Glycemic control HbA1c	Compared to a low fat-diet, there is a decrease in HbA1c in MD (−0.32, 95% −0.53, −0.11) and LC (−0.35, 95% −0.56, −0.14), and a decrease of glycemia in MD (−0.59 mmol/l, 95% −1.13, −0.04).
Emadian, A.; 2015	SR 11 RCTs	MD, Vegan diet, Low glycemic index diet	Overweight adults (IMC ≥25 kg/m^2^) and T2D	>6 months	Low medication control Low adherence	Glycemic control HbA1c	MD, vegan, and low glycemic index diets improve HbA1c levels.
Huo, R.; 2015	MA 9 RCTs	MD	Adults with T2D. *n* = 1178	1 month–4 years	5 low quality studies and 4 high quality. No publication bias for HOMA (Begg’s test), but for HbA1c (p = 0.001, Egger’s test)	Glycemic control HbA1c, insulin, HOMA	Compared to the control group, MD decreased HbA1c (median difference −0.30; 95% CI −0.46, −0.14), glucose (−0.72 mmol/l; CI −1.24, −0.21), and baseline insulin (−0.55 μU/ml; CI −0.81, −0.29).
Carter, P.; 2014	SR, MA 8 RCTs	MD, Paleo diet, Control diet	Overweight and/or high CV risk and/or T2D *n* = 2789	2–12 months	Few studies	Glycemic control HbA1c, insulin.	MD decreased HbA1c compared to the control group, but not to the paleo diet. None of the interventions was better than the other in terms of basal glucose.

## Abbreviations

MD	Mediterranean diet
DASH	Dietary Approach to Stop Hypertension
SR	Systematic review
MA	Meta-analysis
RCTs	Randomized clinical trials
	
T2D	Type 2 diabetes
AHEI	Alternative healthy eating index
CV	Cardiovascular
LC	Low-carbohydrate diet
HbA1c	Glycosylated hemoglobin
HOMA	Insulin resistance index
